# Supplementary material for: Functional Analysis of Two Affinity cAMP Phosphodiesterases in the Nematode-Trapping Fungus Arthrobotrys oligospora
Source: Pathogens. 2022 Mar 26;11(4):405. doi: 10.3390/pathogens11040405 (PMC9026129; doi:10.3390/pathogens11040405)
Supplement: Supplementary file 1 [file pathogens-11-00405-s001.zip › pathogens-1643891-supplementary.pdf]

Supporting Information

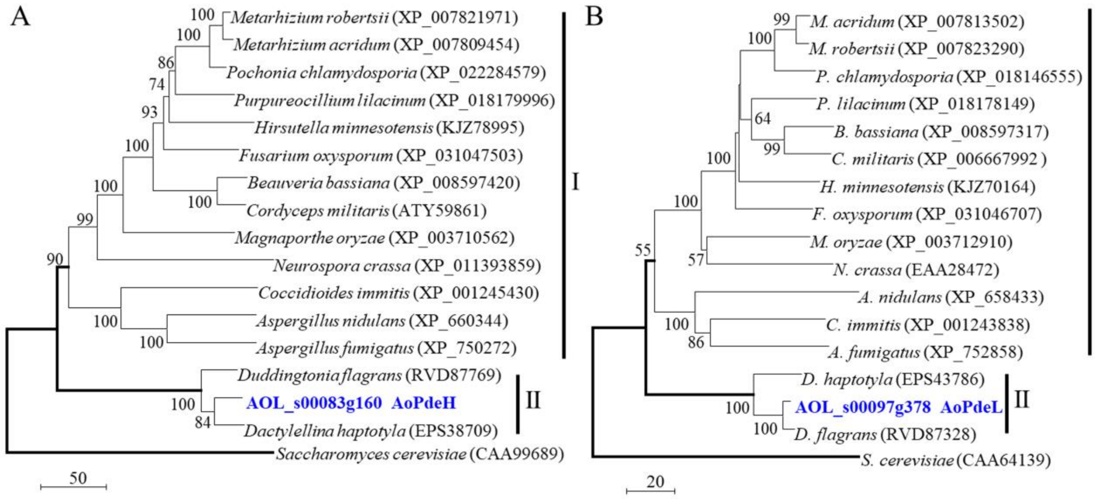

Figure S1. Phylogenetic tree analysis based on the amino acid sequences of PdeH and PdeL homologous proteins from different fungi. (A) Phylogenetic tree of PdeH orthologs from various fungi. (B) Phylogenetic tree of PdeL orthologs from various fungi. GenBank accession numbers are provided in brackets. The amino acid sequences of PdeH and PdeL proteins were aligned with ClustalX version 1.83, and MEGA 7 was used to construct a neighbor-joining tree, including bootstrap analysis with 1,000 replicates.

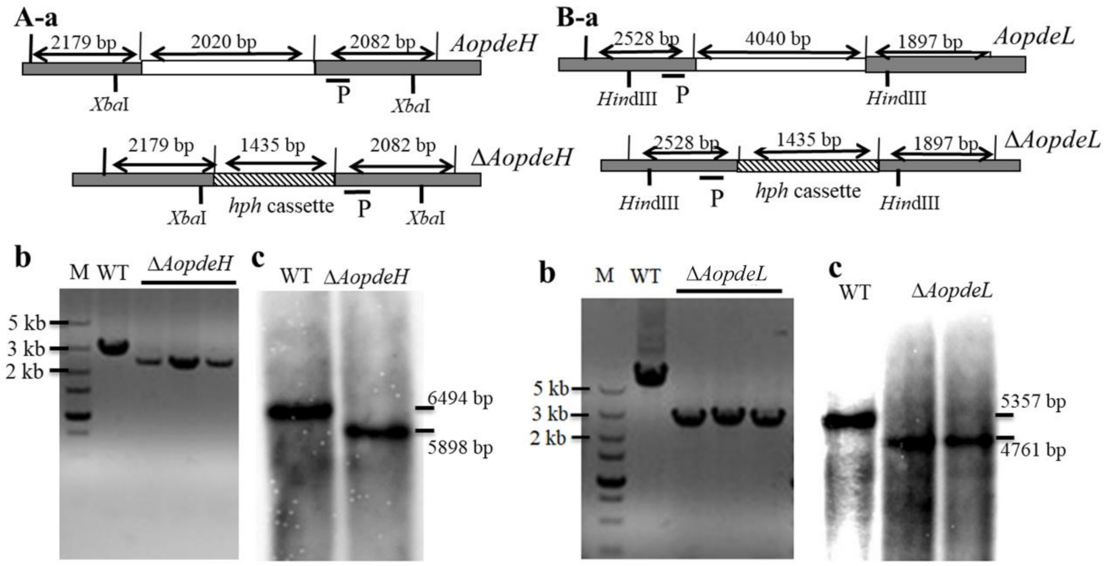

Figure S2. Deletion of *AopdeH* and *AopdeL* in *A. oligospora*. (A) Deletion of *AopdeH* and verification of transformants. (A-a) The diagrammatic sketch of homologous recombination of *AopdeH* and the homologous flanks of the target gene. (**A-b**) *AopdeH*-deleted transformants were confirmed by PCR amplification. M, DNA marker; WT, Wild-type strain; Δ*AopdeH*, transformants. (**A-c**) Southern blotting analysis of wild-type (WT) and transformant (Δ*AopdeH*). **(**B) Deletion of *AopdeL* and verification of transformants. (B-a) The diagrammatic sketch of homologous recombination of *AopdeL* and the homologous flanks of the target gene. (B-b) *AopdeL*-deleted transformants were confirmed by PCR amplification. M, DNA marker; WT, Wild-type strain; Δ*AopdeL*, transformants. (**B-c**) Southern blotting analysis of wild-type (WT) and transformants (Δ*AopdeL*).

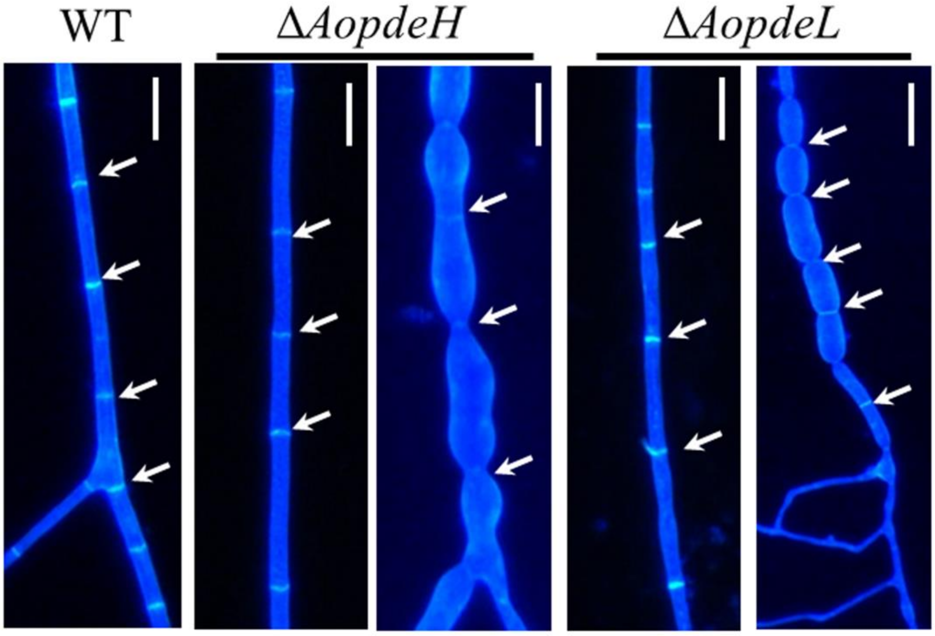

Figure S3. Comparison of the hyphal septum between WT and mutants. The hyphal septa of the WT and mutants were stained with 20 μg/mL calcofluor white (CFW). Arrow, hyphal septum. Bar = 10 μm.

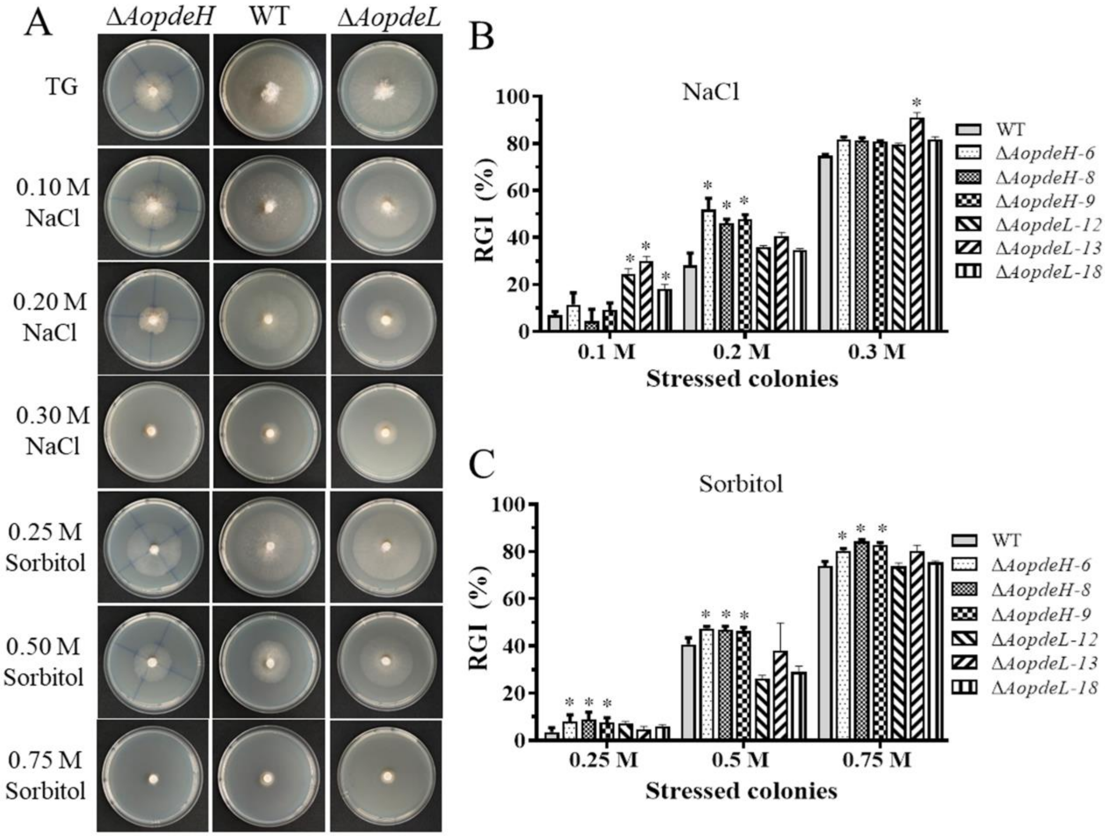

Figure S4. Comparison of osmotic stress responses between WT and mutants. (A) Colonial morphology of fungal strains under osmotic stress. (B) and (C) Relative growth inhibition (RGI) of fungal colonies after being grown for five days at 28 °C on TG plates supplemented with different concentrations of NaCl and sorbitol, respectively. An asterisk indicates a significant difference between mutant and the WT strain (Tukey's HSD, $p < 0.05$).

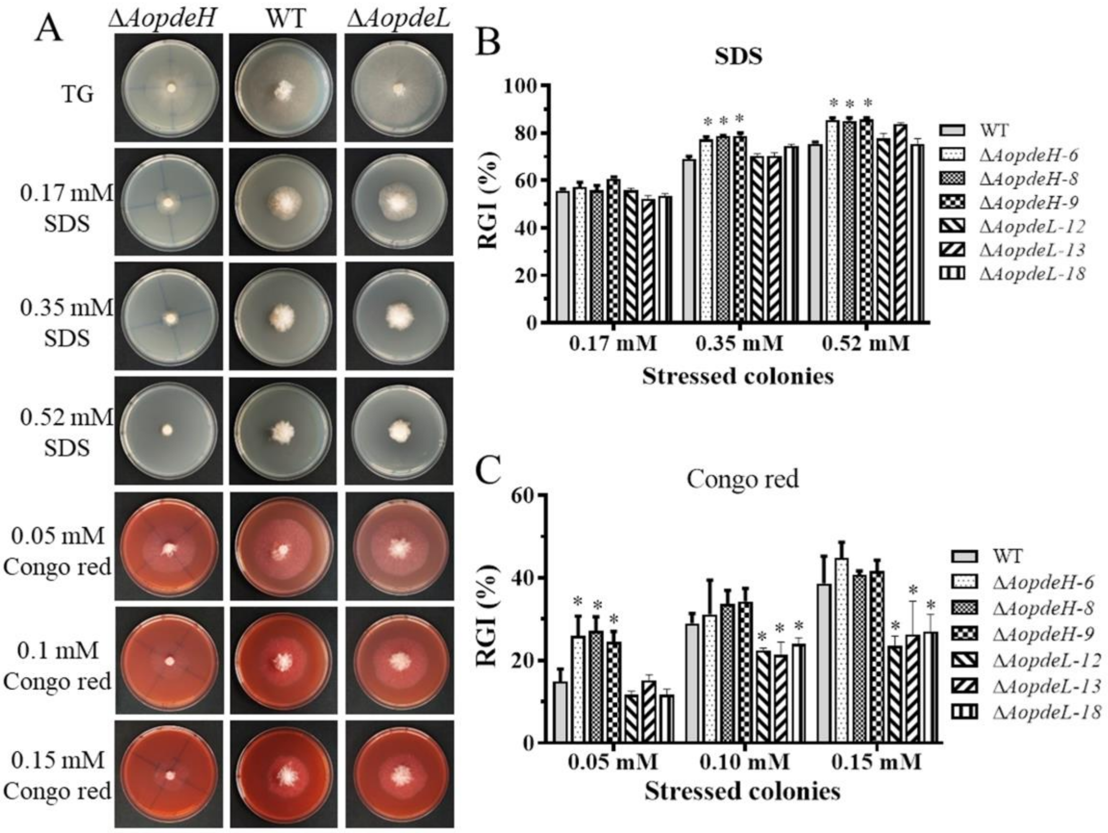

Figure S5. Comparison of cell wall stress agent responses between WT and mutants. (A) Colonial morphology of fungal strains under cell wall stress agents. (B) and (C) Relative growth inhibition (RGI) of fungal colonies after being grown for five days at 28 °C on TG plates supplemented with different concentrations of SDS and Congo red, respectively. An asterisk indicates a significant difference between mutant and the WT strain (Tukey's HSD, $p < 0.05$).

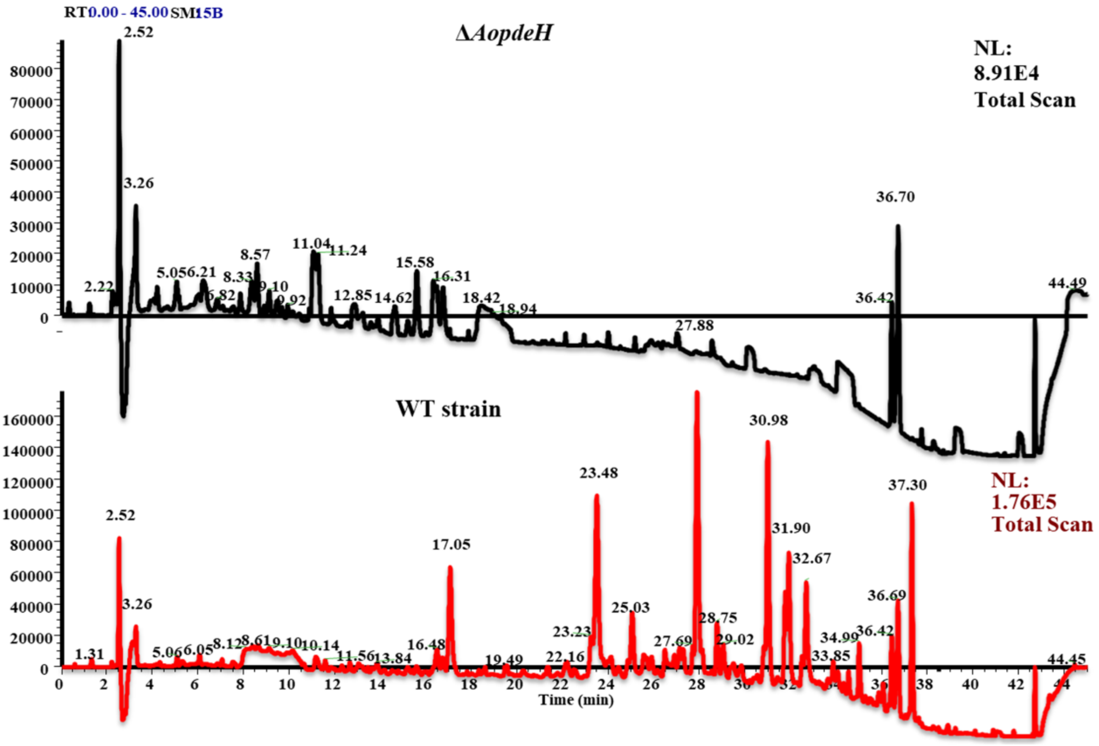

Figure S6. Comparison of high-performance liquid chromatography profiles of the WT and Δ*AopdeH* mutant strains.

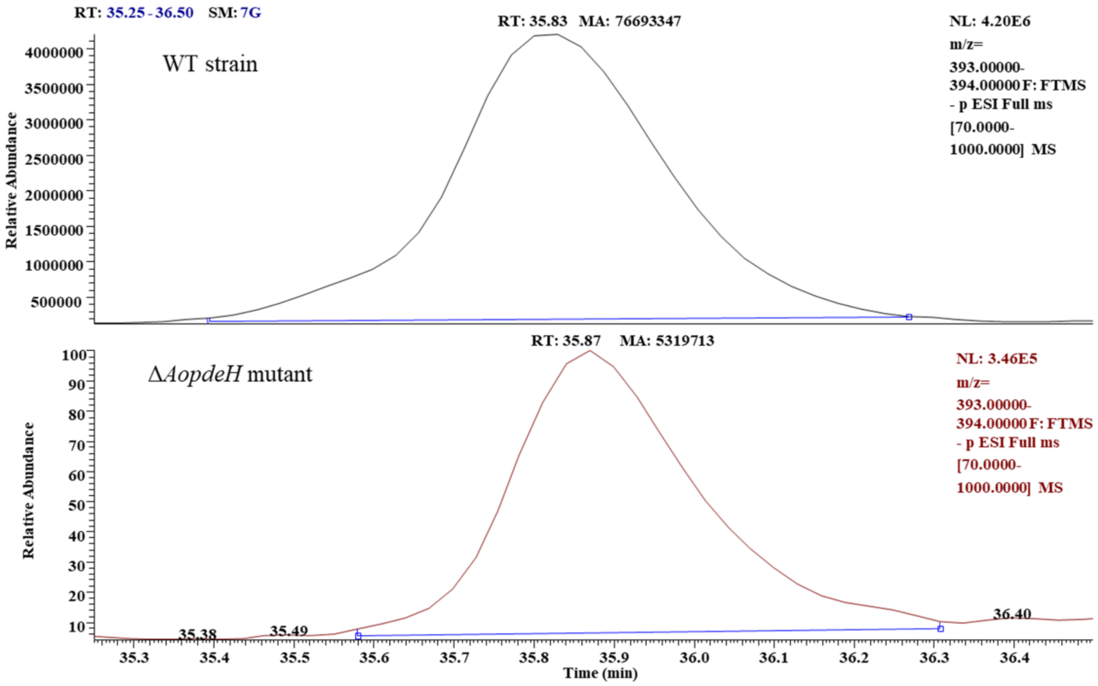

Figure S7. Comparison of the relative peak area of arthrobotrisins between WT and Δ*AopdeH* mutant strains.

Table S1. The sequence similarity between AoPdeH/AoPdeL and orthologs from different fungi. The sequence similarity of orthologous PdeH/PdeL was analyzed using DNAman software.

Species	Simialrity to AoPdeH (%)	Simialrity to AoPdeL (%)	Grounp
Arthrobotrys oligospora	100	100	
Duddingtonia flagrans	89.9	93.3	NT fungi
Dactylellina haptotyla	89.8	72.7	
Neurospora crassa	31.7	34.3	
Aspergillus nidulans	31.5	30.9	
Aspergillus fumigatus	32.8	38.4	
Purpureocillium lilacinum	34.8	41.5	
Hirsutella minnesotensis	34.4	43.1	
Pochonia chlamydosporia	34.8	42.1	Other
Metarhizium robertsii	33.5	43.2	filamentous fungi
Metarhizium acridum	32.9	42.7	
Beauveria bassiana	33.9	41.7	
Cordyceps militaris	32.9	39.5	
Fusarium oxysporum	34.6	42.9	
Magnaporthe oryzae	34.8	40.6	
Coccidioides immitis	32.2	39.4	
Saccharomyces cerevisiae	19.0	28.5	yeast

Table S2. Comparison of the differential compounds-metabolic pathways between the WT and Δ*AopdeH* mutant strains.

Compounds-metabolic pathways	upregulated	downregulated

Superpathway of aromatic compound degradation via 2-oxopent-4-enoate	4	39
Anaerobic aromatic compound degradation (Thauera aromatica)	4	27
Superpathway of aerobic toluene degradation	2	28
Superpathway of aromatic compound degradation via 3-oxoadipate	4	21
Superpathway of chorismate metabolism	4	15
Toluene degradation IV (aerobic) (via catechol)	2	23
Naphthalene degradation to acetyl-CoA	2	23
Mandelate degradation to acetyl-CoA	2	14
Toluene degradation III (aerobic) (via p-cresol)	2	20
Superpathway of aromatic amino acid biosynthesis	4	10
Superpathway of 4-hydroxybenzoate biosynthesis (yeast)	2	13
L-tyrosine degradation IV (to 4-methylphenol)	2	14
Superpathway of scopolin and esculin biosynthesis	2	10
Benzoate biosynthesis I (CoA-dependent, ss-oxidative)	2	14
Superpathway of rosmarinic acid biosynthesis	2	10
Meta cleavage pathway of aromatic compounds	2	8
Superpathway of L-lysine degradation	2	1
Novobiocin biosynthesis	2	9
Toluene degradation I (aerobic) (via o-cresol)	0	12
Other	13	114
Not set	84	1715

Table S3. List of primers used for gene disruption in this study.

Primers	Sequence (5′-3′)	Description
83g160-5F	GTAACGCCAGGGTTTTCCCAGTCACGACGGAAATGGCTCCCGTGACTT-3	Amplify the *AopdeH* gene 5′ flank
83g160-5R	ATCCACTTAACGTTACTGAAATCTCCAACTCAGGCTCAACAACACCGT-3	
83g160-3F	CTCCTTCAATATCATCTTCTGTCTCCGACAGTATCGCCTCTTCGTTGTCC-3	Amplify the *AopdeH* gene 3′ flank
83g160-3R	GCGGATAACAATTTCACACAGGAAACAGCCCTATTACTGGACCCGCATT-3	
97g378-5F	GTAACGCCAGGGTTTTCCCAGTCACGACGTTCCCATCCTTATCGGTCTC-3	Amplify the *AopdeL* gene 5′ flank
97g378-5R	ATCCACTTAACGTTACTGAAATCTCCAACATCCGTCGTTGTCCCCTTA-3	
97g378-3F	CTCCTTCAATATCATCTTCTGTCTCCGACGGGAGAAAAGGCGGGATTA-3	Amplify the *AopdeL* gene 3′ flank
97g378-3R	GCGGATAACAATTTCACACAGGAAACAGCGGATAGCCGAGACTGACCCA-3	
hphF	GTCGGAGACAGAAGATGATATTGAAGGAGC	Amplify the *hph* cassette

hphR	GTTGGAGATTTCAGTAACGTTAAGTGGAT	
83g160-YZF	GTCAGGGCAACGGAGTGTAAA	Verify the transformants for *AopdeH* gene
83g160-YZR	GGAATAGCATCATCGGTAGGA	
97g378-YZF	GCTTGCTGATAATCTTTCCTCG	Verify the transformants for *AopdeL* gene
97g378-YZR	GAACCAACAACTCCGCATAACT	
83g160-TZF	GTCAGGGCAACGGAGTGTAAA	Make Southern blotting probe for *AopdeH* gene
83g160-TZR	GACGCACAGTAACGACAACCATA	
97g378-TZF	GAGTTATGCGGAGTTGTTGGT	Make Southern blotting probe for *AopdeL* gene
97g378-TZR	ACTGGAGGGTTGCTAGAAAGA	

Table S4. List of RT-PCR primers used in this study.

Sporulation-related genes	Sequence (5′-3′)
AOL_s00007g157 (*flbC*)	157-5F-CTCTCCGGCAAAGACAATCG<br>157-3R-GTCGACTGAGGATAGTAGCT
AOL_s00043g361 (*fluG*)	361-5F-GATTCCAGTCCCGTGAATTC<br>361-3R-GCTAAGGAGAGGATGGGCAT
AOL_s00054g700 (*vosA*)	700-5F- CAAACCACCCACCACCAAAT<br>700-3R-GGATGGACAGGAGAAGGACC
AOL_s00054g811 (*velB*)	811-5F- ATTCCGCAACTTCTCCCTCA<br>811-3R- GGCATGTTTGGATTCTGGGG
AOL_s00080g63 (*abaA*)	63-5F-AACTTTATGCGCCTTGTCGT<br>63-3R-TTGGCTAGGTGGTCTGTACG
AOL_s00083g487 (*lreA*)	487-5F-CCCTCCATACAGTCAGTGCTA<br>487-3R-CCCTACCCACCAAGATGATAC
AOL_s00080g93 (*lreB*)	93-5F-CGAAGAGCAAGAAGAACAAGC<br>93-3R-AGATGGGTATGGGATAGAGGG
AOL_s00215g893 (*aspB*)	893-5F-ATACCGCCAACACCCTCTAC<br>893-3R-AACCATCTTCATCTCGGCCT
β-tubulin gene<br>AOL_s00076g640 (*tub*)	tub-5F-CCACCTTCGTCGGTAACTC<br>tub-3R-TCGTCCATACCCTCACCAG
